# Reduced Graphene Oxide Embedded with MQ Silicone Resin Nano-Aggregates for Silicone Rubber Composites with Enhanced Thermal Conductivity and Mechanical Performance

**DOI:** 10.3390/polym10111254

**Published:** 2018-11-12

**Authors:** Weijie Liang, Xin Ge, Jianfang Ge, Tiehu Li, Tingkai Zhao, Xunjun Chen, Yaozhen Song, Yingde Cui, Muhammad Khan, Jianye Ji, Xiaoyan Pang, Ruoling Liu

**Affiliations:** 1 Shaanxi Engineering Laboratory of Graphene New Carbon Materials and Applications, School of Materials Science and Engineering, Northwestern Polytechnical University, Xi’an 710072, China;ztk-xjtu@163.com (T.Z.); mkhan@mail.nwpu.edu.cn (M.K.); 2 Guangdong Engineering Research Center of Silicone Electronic Fine Chemicals, College of Chemistry and Chemical Engineering, Zhongkai University of Agriculture and Engineering, Guangzhou 510225, China; 18011779939@163.com (X.G.); cxj.qiao@163.com (X.C.); yzsong@zhku.edu.cn (Y.S.); jjyjasonky@163.com (J.J.); shelly_pxy@163.com (X.P.); liuruoling2018@163.com (R.L.); 3 Guangzhou Vocational College of Science and Technology, Guangzhou 510550, China; 13602880087@139.com

**Keywords:** reduced graphene oxide, MQ silicone resin, silicone rubber composites, thermal conductivity, mechanical properties

## Abstract

With developments of the electronics industry, more components are being included in electronic devices, which has led to challenges in thermal management. Using reduced graphene oxide embedded with MQ silicone resin (RGO/MQ) nano-aggregates as the composite filler and silicone rubber (SR) as the matrix, a simple approach is designed to prepare RGO/MQ/SR composites. Reduced graphene oxide (RGO) was first used as a substrate for the growth of MQ silicone resin by hybridization, forming sandwich-like micro structured RGO/MQ nano-aggregates successfully. Then, RGO/MQ was integrated into α,ω-dihydroxylpolydimethylsiloxane based on the in situ solvent-free blending method, followed by condensation and vulcanization, fabricating the final RGO/MQ/SR composites. The effective strategy could enhance the adaptability between graphene and silicone matrix under external stimuli at room temperature by embedding nanoscale MQ into the interface of graphene/silicone as the buffer layer. Obvious improvements were found in both thermal conductivity and mechanical properties due to excellent dispersion and interfacial compatibility of RGO/MQ in the host materials. These attractive results suggest that this RGO/MQ/SR composite has potential as a thermal interface material for heat dissipation applications.

## 1. Introduction

Silicone rubber has received considerable attention because of unique properties, such as the excellent environmental adaptivity of polydimethylsiloxane within a broad temperature range, resistance to oxidative degradation, low toxicity and high chemical stability [1,2,3]. Accordingly, it has been widely applied in a variety of settings, such as the aerospace industry, electronics, medical treatment and commodity manufacturing. Among most applications, the high performance of silicone rubber mainly relies on the addition of functional fillers [4,5,6,7,8]. Therefore, it is important to choose appropriate fillers to cater to the specific application requirements of silicone rubber. In recent years, much research interest has been paid to introducing composite fillers to improve the specific performance of silicone rubber [9,10,11,12]. However, uneven dispersion of fillers is a significant challenge to filling and enhancing composites.

Graphene has attracted extensive interest since it was first exfoliated in 2004 [13,14]. Due to its two-dimensional (2D) characteristics with a monolayer of carbon atoms all sp^2^ bonded in a hybridized structure, it exhibits the attractive properties of a large theoretical specific surface area (2675 m^2^·g^−1^), excellent chemical stability, high electrical and thermal conductivity and outstanding mechanical strength [15]. Thus, it is utilized in abroad range of potential applications, including chemical sensors, energy storage and conversion, thermal interface materials and structure materials. Recently, silicone rubber filled with graphene has been reported [16,17,18,19,20]. The incorporation of graphene into silicone rubber has shown enhanced microwave absorption properties, electrical conductivity, energy storage, heat conduction and mechanical performance. However, the optimal enhancement in graphene-based silicone rubber has not been fully achieved due to insufficient dispersion and poor interfacial compatibility. Hence, it is necessary to modify graphene to achieve high dispersion and strong interface interactions with α,ω-dihydroxyl polydimethylsiloxane (PDMS) in the matrix.

MQ silicone resin (MQ) possesses a double-layer compact globular structure and is an important commercial silicone material that consists of a single functional siloxane chain unit (R_3_SiO_1/2_, i.e., M) and four functional siloxane chain units (SiO_4/2_, i.e., Q) [21]. It has a host of excellent properties, such as hydrolysis resistance, antifriction, weathering and ageing resistance, film-forming ability, and radiation resistance, making it suitable for a wide range of applications (e.g., pressure-sensitive adhesives, encapsulations, coatings and personal care products). Given the good compatibility between MQ and polysiloxane, MQ is widely used in industrial silicone applications as a reinforcing filler for elastomers due to its silica content and macroscopic particle-like structure. Although a lot of application studies of MQ are reported in literature [22,23,24], there are few reports on silicone rubber filled with combinations of MQ and other kinds of fillers [25,26].

Combining advantages of both graphene and MQ may be an absorbing and promising alternative to fabricating high-performance silicone rubber. There are no related reports on silicone rubber (SR) composites using graphene/MQ nanocomposite as functional fillers. In this work, reduced graphene oxide embedded with MQ (RGO/MQ) nano-aggregates was fabricated by hybridization of MQ nanospheres on the surface of reduced graphene oxide (RGO). The detailed synthesis route, as well as the involved morphology and structure of the as-prepared RGO/MQ, are presented. The RGO/MQ was incorporated into silicone rubber matrix to form RGO/MQ/SR composites, and performance relating to thermal conductivity and mechanical strength of composites was investigated. The straightforward strategy for preparing RGO/MQ/SR composite does not only make full use of the compatibility between MQ and silicone rubber to improve dispersion and interfacial compatibility between graphene and silicone rubber, but it also ensures full use of RGO/MQ to achieve better thermal conductivity and mechanical properties of composites. The fabricated RGO/MQ/SR composite showed potential application in thermal interface materials. The impact of RGO/MQ on the other properties of silicone rubber composites will be explored systematically in a later study.

## 2. Materials and Methods

### 2.1. Materials

Sulfuric acid (analytical purity, 98%) was provided by Tianjin Third Chemical Factory (Tianjin, China). Anhydrous ethanol (EtOH, AR), hydrochloric acid (HCl, AR), toluene (AR), sodium nitrate (NaNO_3_, AR), potassium permanganate (KMnO_4_, AR) and hydrogen peroxide (H_2_O_2_, AR) were purchased from Damao Chemical Reagent Factory (Tianjin, China). Hexamethyldisiloxane (MM, industrial grade), tetraethyl orthosilicate (TEOS, industrial grade), methyltrimethoxylsilane (MTMS, industrial grade) and dibutyltindilaurate (DBTDL, industrial grade) were purchased from Hangzhou Guibao Chemical Co., Ltd. (Hangzhou, China). PDMS (industrial grade) with viscosities of 20 Pa·s was produced by Foshan Lin Si silicone Materials Co., Ltd. (Foshan, China). Flake graphite was supplied by Qingdao Dongkai Graphite Co., Ltd. (Qingdao, China).

### 2.2. Synthesis of RGO

Graphene oxide (GO) was prepared by modified Hummers method. Firstly, 5 g flake graphite and 5 g NaNO_3_ mixed with 250 mL 98 wt % H_2_SO_4_ were stirred in a round bottom three-neck flask in ice water bath. Then, 30 g KMnO_4_ was added slowly to the solution maintainedat about 0 °C to avoid the risk of explosion caused by the redox reaction. The reaction was maintained at this temperature for 1 h with continuous agitation. The flask with the mixture was transferred to the oil bath at 35 ± 5 °C, stirring for 1 h. Afterwards, the temperature of the oil bath was increased to 95 ± 5 °C and 400 mL deionized water was decanted into the solution gradually. After 30 min, 1000 mL deionized water was poured into the flask. The solution changed to a luminous yellow color during dropwise addition of 30 mL H_2_O_2_ (volume ratio: 35%). The resulting sample was washed with deionized water and ethanol until natural pH value and filtered over a microfiltration membrane (0.45 μm). GO was dried by freeze-drying process (−55 °C for 12 h) and ground by ball grinding technology. Then, GO powders were positioned in a quartz tube reactor under a flow of argon at 600 °C. After thermal reduction, RGO was collected for further application.

### 2.3. Synthesis of MQ

MQ was synthesized by a hydrolysis-polycondensation method. Briefly, first, 71.3 g MM mixed with the mixtures of 6.3 mL EtOH, 38.2 mL H_2_O and 5.9 mL HCl were stirred by power basic stirrer at room temperature for 30 min. Then the temperature of the oil bath was increased to 60 °C and meanwhile 115.5 g TEOS was added into the solution drop by drop. The reaction under stirring and refluxing was maintained at this temperature for 3 h. After the reaction, 50 mL toluene was poured into the solution to extract MQ. The MQ-toluene solution was washed with deionized water until neutral. Finally, MQ was obtained via decompression distillation and drying process.

### 2.4. Synthesis of RGO/MQ

The RGO/MQ was directly fabricated by hybridization. The MQ was firstly dissolved into anhydrous ethanol ultrasonically for 10 min at room temperature. Then the RGO was added into the solution by ultrasonic dispersion for 2 h. The mass ratio of MQ and RGO was 10:1. After forming homogeneous dispersion, the distilled water was dripped into it slowly under strong agitation in order to promote the growth of MQ on the surface of graphene. Some gel appeared in the mixture. Eventually, the mixed system yielded a slurry. Afterwards, the slurry was stirred constantly for 1 h. Subsequently, it was treated by vacuum suction filtration to obtain wet RGO/MQ. Finally, RGO/MQ was dried by vacuum drying at 60 °C for 24 h.

### 2.5. Preparation of RGO/MQ/SRComposites

**Figure 1:**
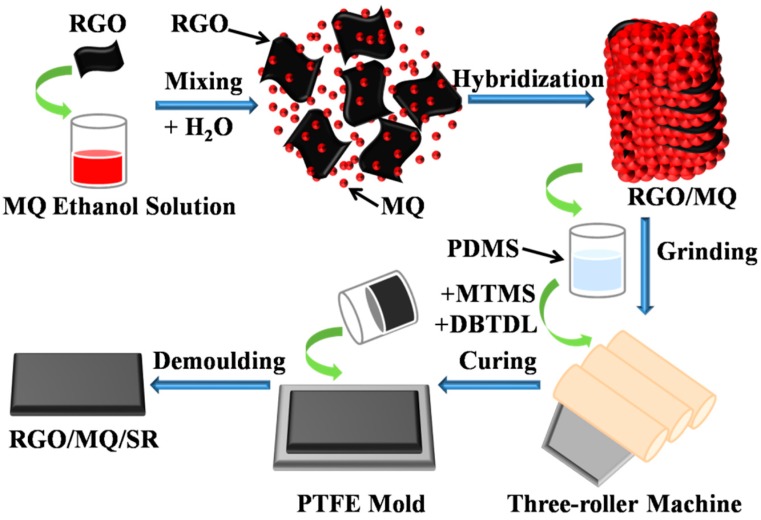
Scheme for the preparation of RGO/MQ/SR composites.

Firstly, PDMS (100.00 g) was compounded with the RGO/MQ (0, 5, 10, 15, 20, or 25 g) by three-roller machine. Then, the crosslinking agent, MTMS (2.5 g), was added and mixed. After that, the catalyst, DBTDL (0.5 g), was incorporated into the mixture. Having been homogenized, the mixture was cast into an open polytetrafluoroethylene (PTFE) mold to cure for more than 24 h at room temperature. The sample of RGO/MQ/SR composites was left standing for 7 days for more sufficient crosslinking before testing. Figure 1 provides the preparation process for RGO/MQ/SR composites. MQ/SR composites and RGO/SR composites were prepared by the same method for comparison.

### 2.6. Characterization

Field emission scanning electron microscope (SEM, SU8220, Hitachi, Tokyo, Japan), X-ray diffraction patterns (XRD, D8 ADVANCE, Bruker AXS, Karlsruhe, Germany), Raman scattering spectrum (LabRAM HR800, HORIBA Scientific, Lat Krabang, Thailand), Fourier transform infrared spectroscopy (FT-IR, Perkin Elmer Spectrum 100, Shelton, DC, USA) was employed for qualitative analyses on the surface morphology, structural characteristics and component composition of MQ, GRO and RGO/MQ. The surface morphologies of cross-sections of RGO/MQ/SR composites and MQ/SR composites were characterized by scanning electron microscopy (EVO18, Carl Zeiss, Jena, Germany). Thermal conductivity of the composites was measured using a universal thermal conductivity meter (TC3000, Xiatech, Xi’an, China), and the dimension of specimens was 50 × 40 × 2 mm^3^. Shore A hardness was examined by LX-A Shore hardness equipment for rubber (Shanghai Precision Instruments Co., Ltd., Shanghai, China). Tensile strength and elongation at break were measured by a universal testing machine (AGS-1, Shimadzu, Kyoto, Japan) according to the GB/T 528–2009 standard.

## 3. Results and Discussion

### 3.1. Morphology and Structure of the RGO/MQ

**Figure 2:**
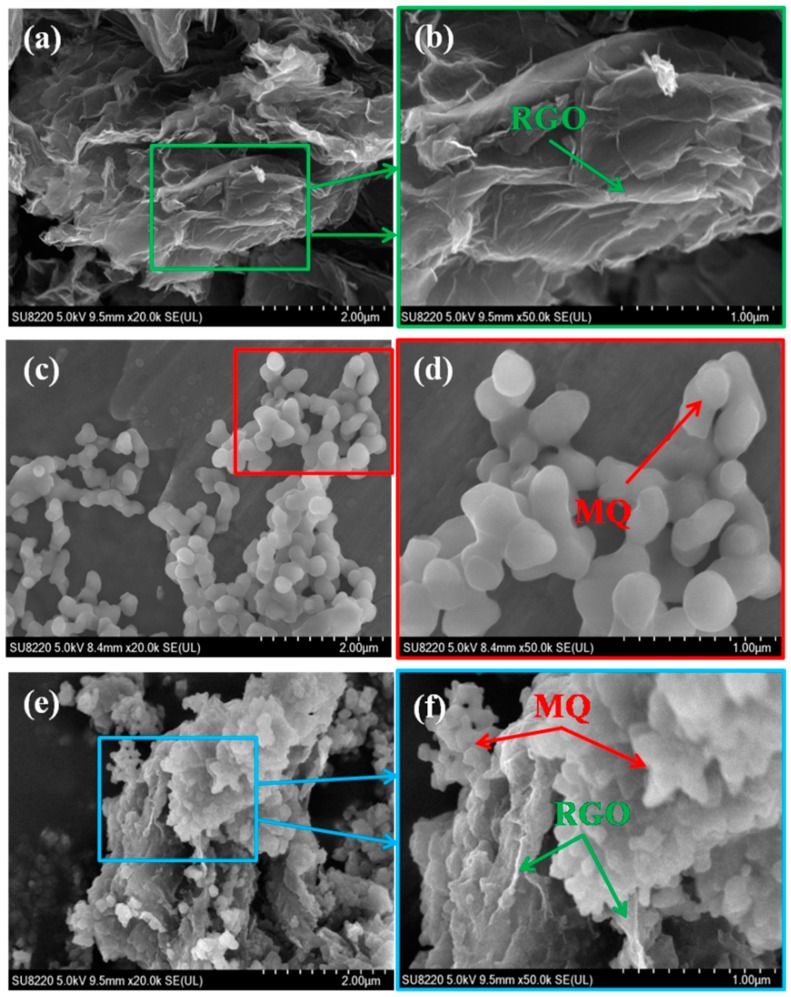
Scanning electron microscopy (SEM) images of (**a**,**b**) RGO, (**c**,**d**) MQ and (**e**,**f**) RGO/MQ at different magnifications.

Figure 2a,c,e shows SEM images of RGO, MQ and RGO/MQ, and Figure 2b,d,f shows respective enlargements. The structure of fluffy and multiple layers is shown in Figure 2a after thermal exfoliation and reduction of GO. Many folds on the surface can be seen clearly in Figure 2b. The edges of RGO partially curl. Figure 2c displays the microstructure of pure MQ in the form of nanoparticles. The morphology of the pristine nanoparticles is spherical. The average diameter of the spherical particles is around 300 nm. Figure 2d is the enlarged picture of Figure 2c, showing the aggregation among nanoparticles. The shapes of the aggregation are different due to randomness of contacting. In Figure 2e it can be seen that agglomerated MQ spheres as the outer modified coating adhere to the surface of lamellar RGO as the internal skeleton. Figure 2f is the high-magnification SEM image of the selected rectangular region of RGO/MQ. In addition to MQ covering RGO directly and densely, some MQ clusters partially fill the lacuna of RGO and lie on the edges of RGO, which demonstrates the existence of the intense interaction between MQ and RGO. This structure shows graphene more efficiently immersed into silicone matrix and provides the channel for heat conduction, as well as increasing the structure stability of composites.

**Figure 3:**
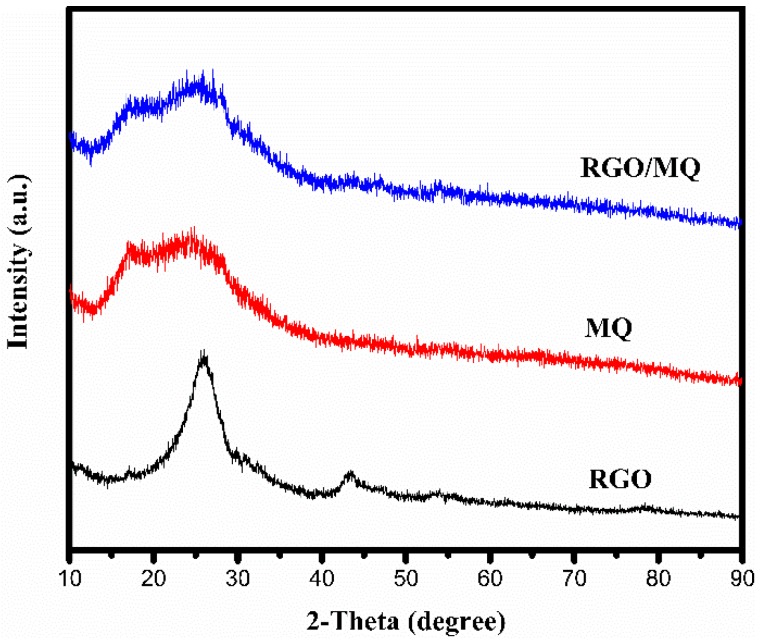
X-ray diffraction (XRD) patterns of RGO, MQ and RGO/MQ.

The XRD patterns of RGO, MQ and RGO/MQ are presented in Figure 3. It can be seen that the RGO have a broad typical diffraction peak at 25.9°, which is typical of multilayered graphene [27], and signifies the graphene phase due to high temperature deoxidization. From the XRD pattern of MQ, obvious characteristic diffraction peaks cannot be found compared to diffuse X-ray peaks. As 2θ ranges from 12.8° to 32.5°, diffuse X-ray peaks are centered at 17.5° and 25.0°, respectively. This indicates MQ crystal structure is amorphous [26]. The RGO/MQ mainly displays diffuse X-ray peaks at 2θ = 12.8–32.5°, which is a little stronger than that of MQ. The existence of weak diffraction peaks at 25.9° and 43.6° of RGO/MQ can be attributed to steric hindrance of MQ. Moreover, no obvious signs of a sharp peak of graphite at 26.6° can be detected in the RGO/MQ [28]. On one hand, this is attributed to RGO after the introduction of MQ. On the other hand, the remarkable face-to-face stacking is broken because of the growth of MQ between the interlayer of RGO, demonstrating that RGO and MQ successfully constitute RGO/MQ.

**Figure 4:**
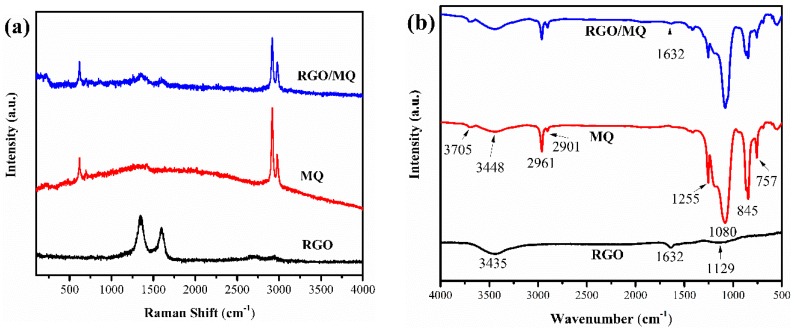
(**a**) Raman spectra of RGO, MQ and RGO/MQ; (**b**) Fourier transform infrared spectroscopy (FT-IR) spectra of RGO, MQ and RGO/MQ.

The Raman spectra of RGO, MQ and RGO/MQ silicone resin are shown in Figure 4a. The RGO curve exhibits two significant peaks in the 1352 and 1595 cm^−1^ regions, which are assigned to the D and G bands of graphene, respectively. The D peak is stronger than the G peak, so the I_D_/I_G_ ratio of graphene is 1.24 for some structure defects and the edge effects of narrow width of graphene sheets [29]. The Raman spectra of MQ has four sharp peaks at 617, 694, 2918 and 2977 cm^−1^, corresponding to stretching vibration of C–Si, symmetrical stretching vibration of SiC_2_, symmetrical stretching vibration of CH_3_ and antisymmetrical stretching vibration of CH_3_, respectively [30]. Although symmetrical stretching vibration and asymmetrical stretching vibration signals of Si–O–Si cannot be easily observed on the Raman spectra of MQ, they can be easily found in the FT-IR spectra of MQ. For the case of RGO/MQ, the reemerging peaks of C–Si, SiC_2_ and CH_3_ and the weaker intensity of D and G bands of graphene compared to RGO demonstrate that MQ is well dispersed on the surface of RGO.

For better understanding of the interaction between RGO and MQ, the FT-IR spectra of RGO, MQ and RGO/MQ are compared in Figure 4b. For the case of RGO, the broad and strong peak at 3435 cm^−1^ is assigned to the characteristic stretching vibration of O–H, while the weak bands at 1632 and 1129 cm^−1^are stretching vibration absorption peaks of C=C and C–O, respectively [31]. The presence of a small number of residual oxygen-containing functional groups indicates that it is hard to remove all the oxygenic groups by thermal reduction. The curve of MQ displays spectrum information of its structure. The small sharp peak centered at 3705 cm^−1^ and the broad absorption peak at 3448 cm^−1^ are due to stretching vibration of free Si–OH and Si–OH with hydrogen-bonded association, respectively [32]. The two sharp peaks at 2961 and 2901 cm^−1^ are related to the C–H asymmetric and symmetric stretching vibrations signal of Si–CH_3_. The bands of Me_3_SiO are 1255, 845, and 757 cm^−1^. The peak with strong and broad shoulders located at 1080 cm^−1^ is asymmetric stretching vibration of Si–O–Si [33]. These peaks all confirm that it is a sample of pure MQ. The spectra of RGO/MQ shows most of the characteristic peaks of MQ due to its sandwich-like microstructure; there is a minor low intensity peak at around 1632 cm^−1^ corresponding to that of RGO, which indicates that MQ and RGO are well combined.

### 3.2. Morphology of the RGO/MQ/SR Composites

**Figure 5:**
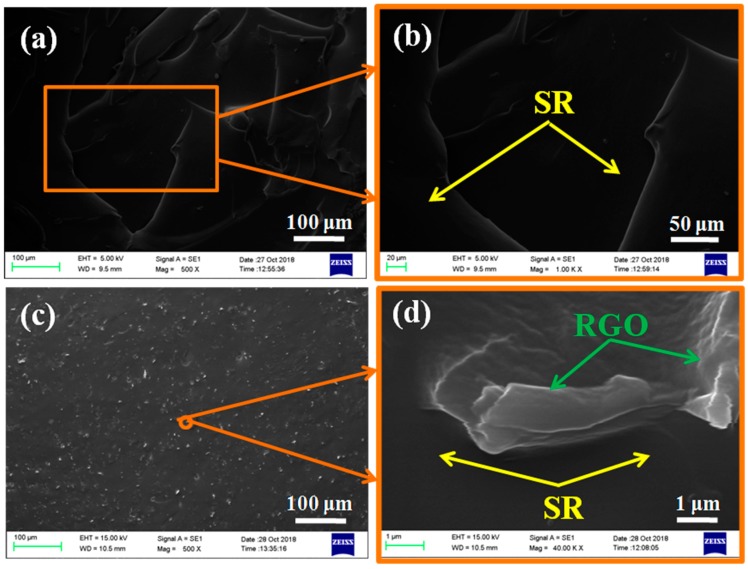
SEM images of (**a**,**b**) MQ/SR composites and (**c**,**d**) RGO/MQ/SR composites at different magnifications.

To fabricate polymer composites, we chose silicone rubber as a flexible matrix and incorporated the RGO/MQ nano-aggregates and pristine MQ by the in situ solvent-free blending method to investigate their morphology. The morphology of composite cross-sectional surfaces was studied by SEM. Figure 5 shows SEM images of MQ/SR composites and RGO/MQ/SR composites. Cracks found on the surface of MQ/SR composites are shown in Figure 5a,b. As a result of the curing reaction of silicone rubber between MQ and PDMS, MQ is not observable in the images. A uniform distribution of small white spots is observable on the surface of RGO/MQ/SR composites as shown in Figure 5c. Figure 5d is the enlarged image of Figure 5c. In Figure 5d, RGO can be observed on the surface of silicone rubber. This observation indicates the small white spots in Figure 5c are RGO. As seen in Figure 5d, there are no apparent signs of microcracks and holes at interfacial regions between graphene and silicone rubber. As observed in Figure 5c,d, few cavities can be found on the surface of silicone rubber filled with RGO/MQ because of the good compatibility and strong interfacial interactions between graphene and polymer matrix, with the help of MQ, according to the basis of the hole theory [34]. Graphene is equally distributed rather than self-aggregated in the internal polymer matrix, which can promote the performance of composites. The result explains that MQ can effectively facilitate the dispersion of graphene.

### 3.3. Thermal Conductivity of the RGO/MQ/SR Composites

**Figure 6:**
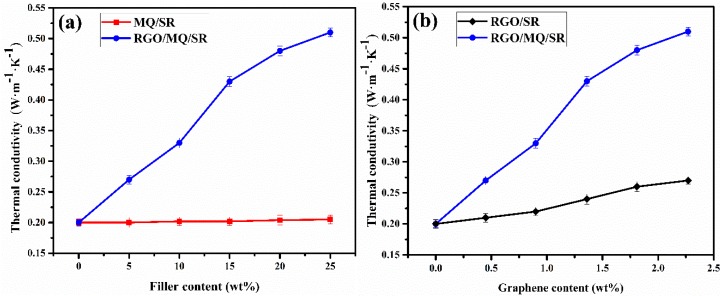
(**a**) Thermal conductivity of MQ/SR composites and RGO/MQ/SR composites with different filler contents; (**b**) thermal conductivity of RGO/MQ/SR composites and RGO/SR composites with different graphene contents.

The result of thermal conductivity tests at room temperature of the composites formed by adding different contents of MQ and RGO/MQ are depicted in Figure 6a. With an increased loading amount of MQ, the thermal conductivity of MQ/SR composites is about 0.20 W·m^−1^·K^−1^ and does not show a substantial change compared to neat silicone rubber without filled MQ. The conduction of heat relies on atomic thermal vibration along the macromolecule chains in the pure silicone rubber. MQ can react with PDMS to improve the density of organic silicone polymeric chain crosslinking, which is not beneficial to heighten the heat transfer capability of phonons of macromolecule chains. Therefore, MQ is not alone suitable as a thermal conductive filler applied to polymer matrices due to its poor thermal conductivity. It is clear that the addition of RGO/MQ results in an increase in thermal conductivity of silicone rubber, which can be attributed to the incorporation of RGO [35]. With content of 5 and 10 wt % RGO/MQ, RGO/MQ/SR composites exhibit a thermal conductivity enhancement of approximately 35% and 65%, respectively. At low RGO/MQ loading, the RGO content of filler was not high enough to accelerate thermal conductivity. This is because RGO does not have good contact with the silicone rubber, but it does contribute to the spreading of phonons in the system. When the RGO/MQ content increases further, the thermal conductivity enhancement of RGO/MQ/SR composites is 115%, 140% and 155% at the content of 15, 20 and 25 wt %, respectively. Owing to the increase in the content of RGO/MQ, more graphene appears in the matrix. An increase in the number of thermal conductive pathways is generated, which is beneficial for forming dense thermal conductive networks. The maximum thermal conductivity of RGO/MQ/SR reaches 0.51 W·m^−1^·K^−1^ in the filler content range, which is significantly higher than that of pure silicone rubber. The heat conduction networks play a major role in the thermal conductivity enhancement due to the effective reduction of the thermal resistance of matrix [19].

Figure 6b represents the thermal conductivity of RGO/SR composites and RGO/MQ/SR composites in accordance with the increments of graphene loadings. The thermal conductivity of RGO/SR composites increases slightly from 0.20 to 0.27 W·m^−1^·K^−1^ with respect to the increasing loading amount of RGO. This result reflects the poor dispersion and weak interaction between RGO and silicone rubber. By contrast, the thermal conductivity of RGO/MQ/SR composites with the same amount of RGO shows a considerable increase. Graphene can form effective thermal conductive pathways in the matrix, accompanying a homogeneous dispersion due to good interfacial compatibility between graphene and matrix assisted by MQ.

**Figure 7:**
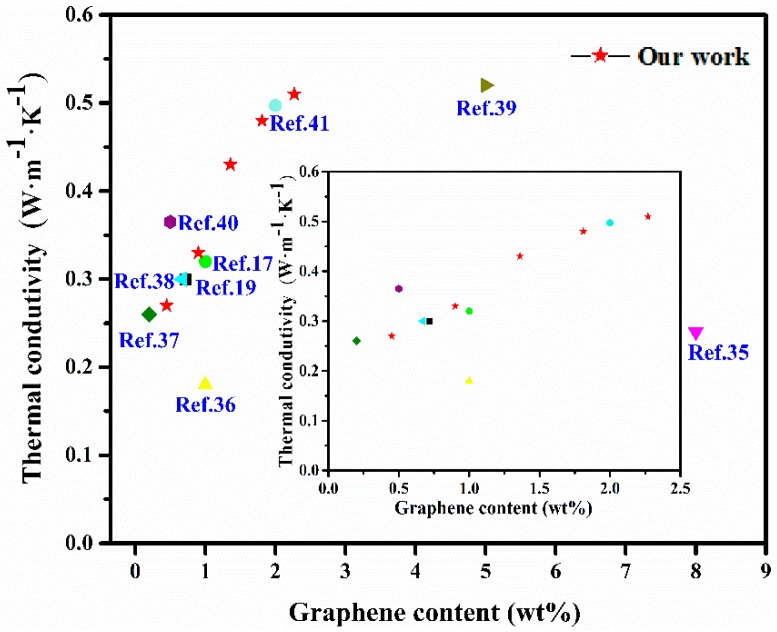
Comparison of thermal conductivity of graphene/silicone rubber composites presented in literature and our work.

In contrast to other previous work, the results of thermal conductivity show greater improvement, shown in Figure 7. Our work shows thermal conductivity of RGO/MQ/SR composites with a lower graphene content are competitive with those of other graphene/silicone rubber composites previously reported in the literature [17,19,35,36,37,38,39,40,41]. The improved thermal conductivity demonstrates that sandwich-like micro structured RGO/MQ can motivate the dispersion of RGO in the matrix which fully utilizes the thermal conductance of graphene. The achievement of RGO/MQ/SR composites can be exploited for thermal interface materials for thermal management.

### 3.4. Mechanical Properties of the RGO/MQ/SR Composites

**Figure 8:**
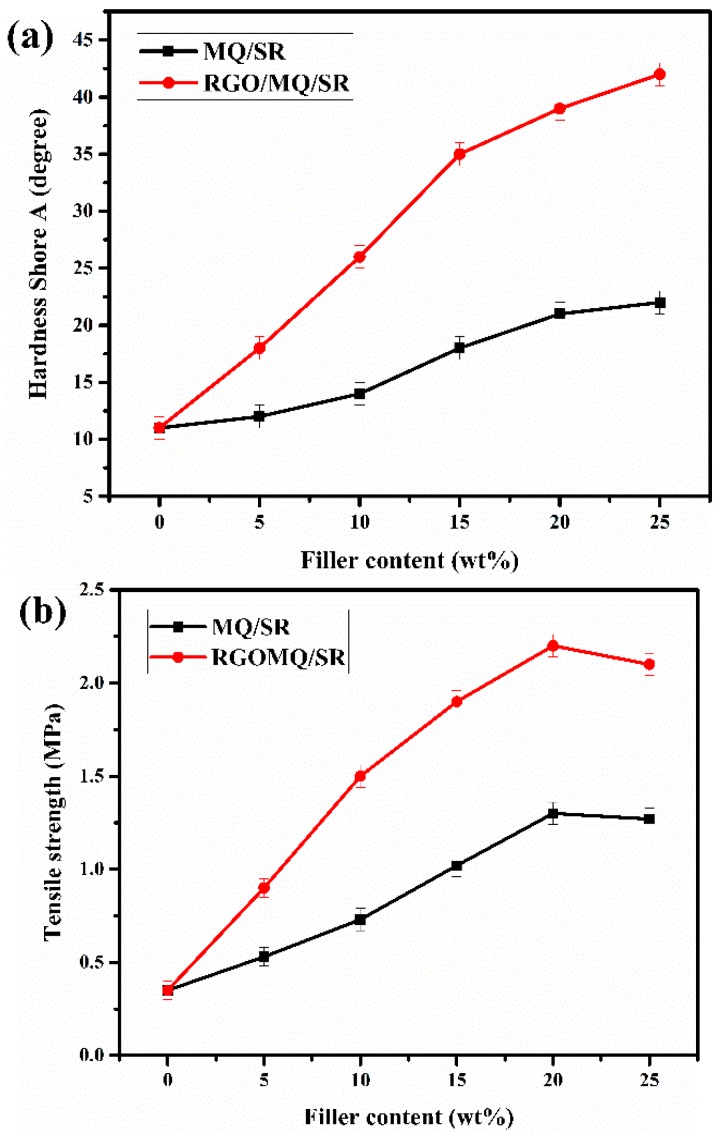

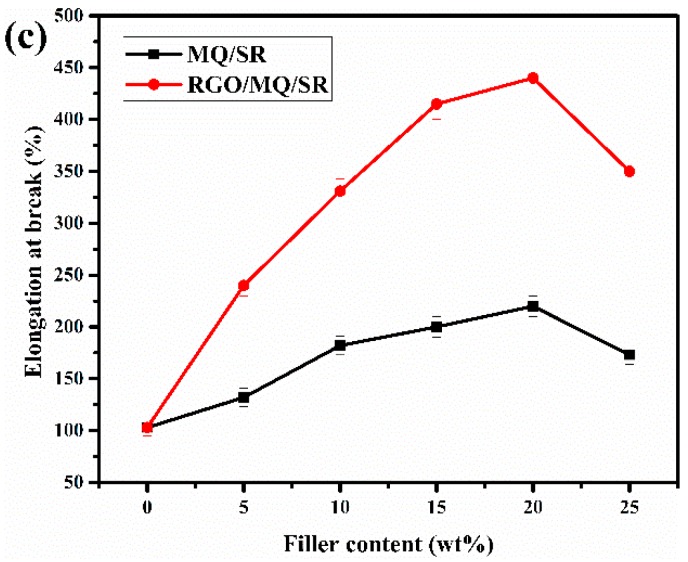
Effects of MQ and RGO/MQ content on (**a**) Shore A hardness, (**b**) tensile strength and (**c**) elongation at break of silicone rubber composites.

To compare the effect of filler type and content, investigations of mechanical properties of MQ/SR and RGO/MQ/SR composites were conducted. Shore A hardness was tested to assess the ability of composites to resist indentation by a hard object. As can be seen from Figure 8a, Shore A hardness of RGO/MQ/SR composites increases substantially with the increase of content of RGO/MQ in the experimental range, and is up to 254% and 281% harder with 20 and 25 wt % addition, respectively, which is much higher than for the corresponding MQ/SR composites. The excellent mechanical strength and very high aspect ratio of RGO, as the framework introduced into MQ, led to greater reinforcement in the resulting composites [42].

Tensile strength values were examined to study the ability of the as-prepared composites to resist permanent deformation and destruction, and the results are shown in Figure 8b. The tensile strength of MQ/SR composites increased with the increment of MQ from 0 to 20 wt %, which can be attributed to the increase in crosslinking density [22] that occurs because the hydroxyl group of MQ can react with the terminal hydroxyl group of PDMS to form a crosslinking network by condensation in silicone rubber. For RGO/MQ/SR composites, the tensile strength value declined after rising to a maximum value of 2.2 MPa (20 wt %), which was much higher than the 1.3 MPa recorded for MQ-filled silicone rubber. When RGO/MQ is integrated into silicone rubber, MQ acts as a bridge between the RGO and rubber matrix so that the compatibility of RGO with polysiloxane is greatly improved. Reducing the agglomeration of the graphene by MQ, RGO can form reinforcing centers through physical entanglement with the chains of PDMS. Moreover, the effective interfacial interactions between RGO and PDMS promote the load transfer from rubber matrix to nanofiller. The synergistic effect between the crosslinking function generated by MQ and PDMS, and physical connection formed by RGO and PDMS, plays an important role in improving tensile strength of RGO/MQ/SR composites. Enhancement of tensile strength decreased at the filling ratio of 25 wt % of RGO/MQ, which might be due to filled RGO/MQ reaching a critical level and RGO beginning agglomeration. The poor dispersion of RGO weakens interaction with PDMS, and so obstructs the reinforcement of RGO/MQ/SR composites.

The elongation at break values was measured to survey the tensile processes before reaching the breaking point of the as-prepared polysiloxane matrices (Figure 8c). The elongation at break of silicone rubber first increases and then decreases with the increasing loading amount of MQ and RGO/MQ. At the same loading amount of filler, the elongation at break of RGO/MQ/SR composites was much greater than that of MQ/SR composites. The elongation at break of the RGO/MQ/SR composites increased from 103% to 440% when the RGO/MQ content rose from 0 to 20 wt %. However, when the RGO/MQ content reached 25 wt %, the elongation at break of RGO/MQ/SR composites decreased to 350%. The decrease results from excessive crosslinking density mainly caused by MQ and insufficient dispersion generated by RGO between fillers and matrixes. Compared with that of pure SR, improvement in the mechanical properties of the filled SR was achieved on account of the packing type [43]. Comparing the fillers of MQ and RGO/MQ, pristine MQ presented the improved mechanical properties of the polysiloxane matrix, and RGO/MQ showed much greater improvement after introducing RGO as the dispersion supporter.

## 4. Conclusions

This study prepared RGO/MQ nano-aggregates by hybridization and then investigated the effect of RGO/MQ and pristine MQ loading on thermal conductivity and mechanical performance of the resulting silicone rubber composites. We found that RGO/MQ exhibited a sandwich-like microstructure. Furthermore, it was revealed that the addition of an optimal ratio of RGO/MQ to SR can simultaneously enhance thermal conductivity and mechanical performance of the resulting SR composites, which can be mainly attributed to excellent distribution of RGO/MQ and strong interfacial compatibility of the composites. The RGO/MQ/SR composites resulting from the addition of 20 wt % RGO/MQ exhibited concurrent improvements in thermal conductivity, Shore A hardness, tensile strength and elongation at break of 140%, 254%, 528%, and 327%, respectively, compared to neat silicone rubber. In light of its solvent-free nature and these outstanding properties, RGO/MQ/SR composite is envisioned to have enormous potential as a thermal interface material in thermal management.
